# Sodium Intakes of US Children and Adults from Foods and Beverages by Location of Origin and by Specific Food Source

**DOI:** 10.3390/nu5061840

**Published:** 2013-05-28

**Authors:** Adam Drewnowski, Colin D. Rehm

**Affiliations:** 1 Université Pierre et Marie Curie—Paris VI, Groupe Hospitalier Pitié-Salpêtrière, 91 boulevard de l’Hôpital, Paris 75013, France; 2 Center for Public Health Nutrition, University of Washington, Box 353410, Seattle, WA 98195, USA; E-Mail: crehm@uw.edu

**Keywords:** dietary sodium, food away from home, fast foods, food source, dietary surveillance, nutrition surveys

## Abstract

Sodium intakes, from foods and beverages, of 22,852 persons in the National Health and Nutrition Examination Surveys (NHANES 2003–2008) were examined by specific food source and by food location of origin. Analyses were based on a single 24-h recall. Separate analyses were conducted for children (6–11 years of age), adolescents (12–19), and adults (20–50 and ≥51 years). Grouping of like foods (e.g., food sources) used a scheme proposed by the National Cancer Institute, which divides foods/beverages into 96 food subgroups (e.g., pizza, yeast breads or cold cuts). Food locations of origin were stores (e.g., grocery, convenience and specialty stores), quick-service restaurant/pizza (QSR), full-service restaurant (FSR), school, or other. Food locations of sodium were also evaluated by race/ethnicity amongst adults. Stores provided between 58.1% and 65.2% of dietary sodium, whereas QSR and FSR together provided between 18.9% and 31.8% depending on age. The proportion of sodium from QSR varied from 10.1% to 19.9%, whereas that from FSR varied from 3.4% to 13.3%. School meals provided 10.4% of sodium for 6–11 year olds and 6.0% for 12–19 year olds. Pizza from QSR, the top away from home food item, provided 5.4% of sodium in adolescents. QSR pizza, chicken, burgers and Mexican dishes combined provided 7.8% of total sodium in adult diets. Most sodium came from foods purchased in stores. Food manufacturers, restaurants, and grocery stores all have a role to play in reducing the amount of sodium in the American diet.

## 1. Introduction

Reducing the sodium content of the American diet is at the top of the public health action agenda [1,2,3,4,5]. Reports from the US Department of Agriculture’s (USDA) Economic Research Service (ERS) have fueled concerns that one significant source of dietary sodium is represented by foods purchased and eaten away from home [6,7,8]. Since such foods can represent up to 32% of total daily calories, their potential impact on sodium consumption and health can be significant [9,10]. The USDA reports have linked food away from home (FAFH) with higher consumption of fats, sugars and salt, and with lower-quality diets overall [6,7,8]. 

In general, FAFH has been equated with foods obtained from or consumed in restaurants, including both fast-food and full-service restaurants [11]. The ERS/USDA has classified meals as FAFH if the majority of calories in that meal, excluding beverages, came from fast-food or full-service restaurants, cafeterias, or taverns [7]. Strictly speaking, the definition of away-from-home foods should encompass all foods that are prepared, purchased, and consumed away from home, including those obtained from schools, workplace cafeterias, vending machines, pre-prepared at grocery stores and from other people. 

Food locations of origin can now be determined much more precisely. Since 2003, the National Health and Nutrition Examination Survey (NHANES) has coded all foods consumed by participants by their location of origin: store, quick-service restaurant/pizza (QSR), full-service restaurant (FSR), school, workplace, vending machine, gift, grown, or other. We used that coding scheme to assign all FAFH into appropriate subcategories. This differentiation of FAFH into subcategories by food location of origin can help inform public policy on ways to improve the quality of the American diet. In particular, the subcategories allow us to estimate sodium consumption from stores *vs.* restaurants, both FSR and QSR.

Specific food sources of sodium can also be identified with greater precision. Dietary intake data from the 2005–2006 NHANES were recently aggregated into 96 mutually exclusive food groups by the National Cancer Institute [12]. This classification scheme for sources of dietary sodium was prominently featured in the 2010 Dietary Guidelines [13]. Food codes representing similar foods—such as the various types of pasta dishes—were combined to provide an indication of how much sodium was provided by that food group to the total diet. Previous analyses have explored the sodium content of processed foods in the United Kingdom [14] and in Australia [15,16].

Combining these two methods allowed for the determination of the primary sources of dietary sodium for children (6–11), adolescents (12–19), and adults (20–50 and ≥51) by food location of origin and by specific food source. Such analyses provide new and unprecedented insight into sodium intakes at home and away from home and can be used to shape and target public health policies for sodium reduction for different age groups.

## 2. Experimental Section

### 2.1. Dietary Intake Databases

Data from three cycles of NHANES for 2003–2004, 2005–2006, and 2007–2008 were used to identify the main sources of dietary sodium by age group, food location of origin, and by specific food source.

Separate analyses were conducted for children (6–11), adolescents (12–19), and for younger (20–50) and older adults (≥51). The 2003-8 NHANES database includes 3033 children; 5432 adolescents, and 14,387 adults for a total of 22,852 persons. The present analyses used Day 1 responses, based on in-person interviews with respondents listing the types and amounts of all food and beverages consumed in the preceding 24-hours. The first recall day was used here since we were most interested in population-level consumption patterns rather than the patterns of individuals. In the evaluation of dietary habits for populations or sub-populations, a single 24-h recall should provide an unbiased estimate of intake at the population-level. For children 6–11 years of age, the child was the primary respondent, but the proxy was present and able to assist. For children 12 years of age and older, the child was the primary source of dietary recall information, but could be assisted by an adult who had knowledge of their diet [17]. 

### 2.2. Food Locations of Origin

For each food or beverage listed, NHANES data provided information on the locations where the food was obtained or purchased (*i.e.*, “food locations”). The primary locations were stores, quick-service restaurants or pizza take-out/delivery (QSR), full-service restaurants (FSR), school and from someone else/gift. Additional food locations were vending machines, other type of cafeterias including workplace, grown or caught (e.g., through gardening or hunting), tavern/bar, or from sporting/cultural/entertainment event (e.g., movie theater or baseball game). Grocery stores, supermarkets, convenience stores, and specialty food stores were not differentiated. The system did not distinguish between foods purchased at stores and prepared at home and store-prepared foods, an increasingly important category. For the present analyses, the primary locations where the foods and beverages were obtained were narrowed to stores, QSR or pizza take-out/delivery, FSR and a combined “other” category. 

The food location information from NHANES was then used to estimate the relative contribution of the sodium content of the US diet by age group and by race/ethnicity by calculating the survey-weighted ratio of the means (referred to elsewhere as the population proportion) [18]. Analyses of food locations by race/ethnicity were adjusted for age group to account for differences in the age distribution by race/ethnicity. Race/ethnicity was defined as non-Hispanic white, non-Hispanic black, Mexican-American/other Hispanic and other race/mixed race.

### 2.3. Specific Food Sources

The Food and Nutrient Database for Dietary Studies (FNDDS) provided a detailed description for each food and beverage consumed by NHANES participants [19]. All FNDDS foods were aggregated into eight broad food groups and 96 food subgroups, based on food groups developed by the National Cancer Institute (NCI) [12]. Linking of FNDDS codes with NCI food groups was done by the study authors. These groupings are useful because they show that the food sources that are the highest in sodium may not be the most important sources of dietary sodium on a population-level. Examples of food groups were soda, energy and sports drinks, yeast breads, grain-based desserts, burgers, fried potatoes, pizza, sandwiches, chicken dishes, or mixed Mexican dishes. A list of these food groups can be found in the Appendix.

This food classification scheme was used to estimate the relative contribution of different food sources (specific foods) to sodium intakes by age group. Then, the specific food sources were identified by food location of origin, separately for each age group. For example, such analyses allowed us to distinguish the contribution of pizza from stores *vs.* pizza from QSR to sodium intakes by age or demographic group. The standard error of the mean or proportion was estimated and results are not presented when the relative standard error ([SE/point estimate] × 100) was greater than 40%. Results with a relative standard error between 30% and 39.9% were flagged as being potentially unstable. Because NHANES is a complex sample survey, all analyses reported here were survey-weighted to account for the survey design and reflect the behaviors of the United States population.

## 3. Results

### 3.1. Sodium Intakes by Food Location of Origin

**Figure 1:**
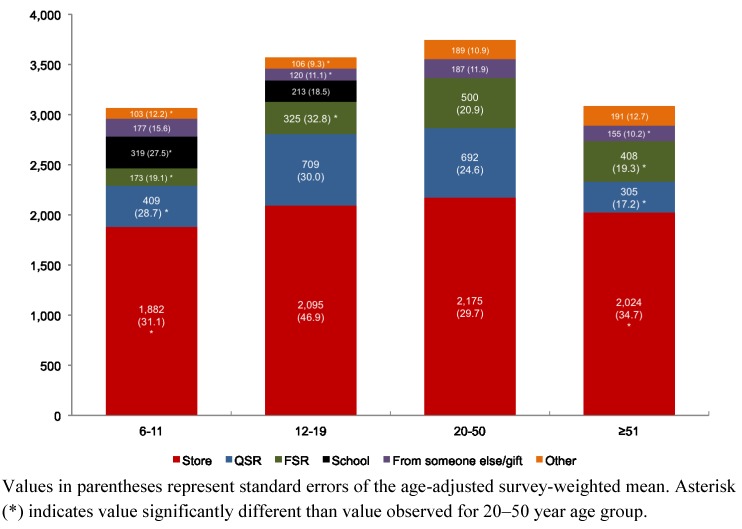
Location of dietary sodium (mg) by age group (year), NHANES 2003–2008.

Data presented in Figure 1 show that both sodium intakes and food locations of origin depended on age. First, sodium intakes increased and then declined with age, as expected due to the previously observed correlation between sodium and energy [3]. Depending on age, stores and restaurants together provided up to 89.9% of sodium in the American diet. Sodium locations of origin also varied sharply as a function of age.

For primary school-aged children (6–11), 61.4% of sodium came from stores, 13.3% from QSR, and 10.4% from school cafeterias. Among adolescents (12–19), 58.7% of sodium came from stores, 19.9% from QSR, 9.1% from FSR, and 6.0% from school meals. For adults aged 20–50 years, 58.1% of sodium came from stores, as compared to 18.5% from QSR and 13.3% from FSR. For older adults (≥51), 65.2% of sodium came from stores, 10.1% from QSR, and 13.3% from FSR. 

Adults, aged 20–50 years, were the group that obtained the highest proportion of dietary sodium (31.8%) from restaurants, both QSR and FSR, followed by the 12–19 year age group (29.0%). Although the overall amount of sodium obtained from restaurants declined after age 50, sodium from QSR tended to be replaced with sodium from FSR.

**Figure 2:**
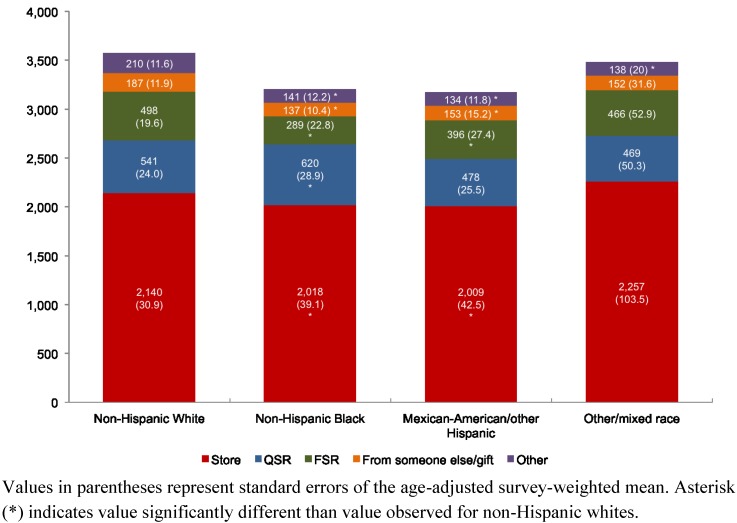
Location of dietary sodium (mg) by race/ethnicity among adults (age ≥20 years), NHANES 2003–2008.

For different race/ethnicity groups, the combined contribution of QSR and FSR to total dietary sodium was between 26.9% and 29.1%. Figure 2 shows that non-Hispanic whites obtained the lowest proportion of sodium from stores (2140 mg/day or 59.8%) as compared to non-Hispanic blacks (2018 mg/day or 63.0%) and Mexican-Americans/other Hispanics (2009 mg/day or 63.4%). Non-Hispanic whites obtained another 541 mg/day (15.1%) from QSR and 498 mg/day (13.9%) from FSR. Non-Hispanic blacks obtained 620 mg/day (19.3%) from QSR and 289 mg/day (9.0%) from FSR. Mexican-Americans/other Hispanics obtained 478 mg/day (15.1%) from QSR and 396 mg/day (12.5%) from FSR.

### 3.2. Sodium Intakes by Specific Food Sources

Table 1 shows the contribution of specific food sources to sodium intakes by age group. Data are presented for sodium as mg/day and % of daily intake for the top 24 food groups for the total population; the remaining specific food groups contributed <2.0% of dietary sodium for each age group.

For children, pizza provided 8.3% of dietary sodium, yeast breads provided 7.9%, pasta and pasta dishes 6.9%, chicken dishes 6.9% and sausage and franks 6.2%. These foods were among the top sources of sodium across all age groups. Among adolescents, the top sources of sodium were pizza (10.3%), yeast breads (7.5%), and pasta (5.2%), followed by chicken and chicken dishes, sausages, and cheese. 

Adults aged 20–50 derived 7.3% of sodium from chicken and dishes, 7.2% from yeast breads and 6.4% from pizza, followed by pasta dishes, sausages and beef. Adults aged ≥51 years derived 9.4% of sodium from bread, 5.6% from chicken, 5.2% from sausage, and 4.9% from beef. Soups accounted for 4.7% of sodium whereas pizza accounted for only 3.0%.

### 3.3. Sodium Intakes by Location of Origin and Specific Food Source

Table 2 shows sodium intakes by purchase location and specific food source for children aged 6–11. The top three sources of sodium came from the store. These were yeast breads (5.7%), pasta dishes (5.5%) and sausage and franks (4.3%). Pizza, the top QSR item, contributed 3.4%, followed by a number of store bought items and then by QSR chicken (2.2%) and QSR burgers (1.5%). 

Table 3 shows sodium intake data for adolescents. The top source of sodium was yeast bread (5.5%), closely followed by QSR pizza (5.4%), store pasta dishes (4.3%), sausage (3.3%) and QSR chicken (3.0%). QSR burgers contributed 2.4% of daily sodium.

Table 4 shows sodium intake data for adults aged 20–50. Store bought yeast bread (5.1%) and pasta (3.7%) were followed by QSR pizza (3.7%) and store bought sausage (3.4%), chicken (2.7%) and beef (2.7%). QSR chicken contributed 2.6% and QSR burgers 1.8%.

Table 5 shows sodium intake data for adults ages ≥51. The top 16 sodium sources came from the store, led by yeast breads (7.2%), sausage (3.6%), soups (3.3%), pasta (2.9%), beef (2.8%), cold cuts (2.8%) and grain-based desserts (2.6%). The top restaurant item was chicken dishes from QSR with 1.4% and chicken dishes from FSR with 1.2%. QSR burgers contributed 1.0%.

**Table 1:** Contribution to dietary sodium by specific food sources by age group, NHANES 2003–2008.

	6–11 years		12–19 years		20–50 years		≥51 years		
Category ^1^	Na [mg] (SE)	% Total (SE)	Na [mg] (SE)	% Total (SE)	Na [mg] (SE)	% Total (SE)	Na [mg] (SE)	% Total (SE)	*p*-value
Pizza	253 (14.4)	8.3 (0.5)	369 (22.9)	10.3 (0.6)	239 (13.8)	6.4 (0.4)	91 (11.2)	3.0 (0.4)	<0.001
Yeast breads	243 (10.2)	7.9 (0.4)	266 (10.0)	7.5 (0.3)	270 (6.7)	7.2 (0.2)	288 (7.4)	9.4 (0.2)	<0.001
Pasta and pasta dishes	213 (16.0)	6.9 (0.5)	187 (12.4)	5.2 (0.3)	181 (9.7)	4.8 (0.2)	121 (8.2)	4.0 (0.3)	<0.001
Chicken and chicken mixed dishes	210 (15.6)	6.9 (0.5)	281 (13)	7.9 (0.3)	274 (8.8)	7.3 (0.2)	170 (6.8)	5.6 (0.2)	<0.001
Sausage, franks, bacon, and ribs	191 (10.7)	6.2 (0.4)	153 (8.1)	4.3 (0.2)	177 (8.7)	4.7 (0.2)	159 (8.3)	5.1 (0.3)	<0.001
Regular cheese	122 (9.0)	4.0 (0.3)	141 (7.1)	3.9 (0.2)	145 (5.3)	3.9 (0.1)	107 (5.1)	3.5 (0.2)	<0.001
Grain-based desserts	116 (7.0)	3.8 (0.2)	111 (3.6)	3.1 (0.1)	108 (3.7)	2.9 (0.1)	114 (4.8)	3.7 (0.1)	<0.001
Ready-to-eat cereals	108 (5.6)	3.5 (0.2)	92 (3.6)	2.6 (0.1)	56 (2.3)	1.5 (0.1)	63 (2.5)	2.1 (0.1)	<0.001
Beef and beef mixed dishes ^3^	106 (10.4)	3.5 (0.3)	149 (8.3)	4.2 (0.2)	174 (6.0)	4.7 (0.2)	149 (7.9)	4.8 (0.3)	<0.001
Mexican mixed dishes	104 (18.9)	3.4 (0.4)	163 (19.4)	4.6 (0.5)	172 (11.5)	4.6 (0.3)	78 (9.2)	2.5 (0.3)	<0.001
Reduced fat milk	98 (4.9)	3.2 (0.2)	70 (4.0)	2.0 (0.1)	42 (2.3)	1.1 (0.1)	39 (1.9)	1.3 (0.1)	<0.001
Soups	93 (9.7)	3.0 (0.3)	107 (10.7)	3.0 (0.3)	127 (6.8)	3.4 (0.2)	143 (9.5)	4.6 (0.3)	<0.001
Potato/corn/other chips	81 (4.4)	2.7 (0.1)	98 (4.8)	2.7 (0.1)	64 (2.6)	1.7 (0.1)	38 (1.8)	1.2 (0.1)	<0.001
Cold cuts	77 (6.7)	2.5 (0.2)	125 (9.6)	3.5 (0.3)	129 (7.0)	3.4 (0.2)	108 (4.9)	3.5 (0.1)	0.004
Pancakes/waffles/French toast	73 (6.8)	2.4 (0.2)	43 (4.8)	1.2 (0.1)	30 (2.7)	0.8 (0.1)	30 (4.3)	1.0 (0.1)	<0.001
Rice and rice mixed dishes	63 (7.2)	2.0 (0.2)	79 (7.8)	2.2 (0.2)	128 (7.7)	3.4 (0.2)	77 (6.5)	2.5 (0.2)	<0.001
Condiments	62 (4.3)	2.0 (0.1)	92 (7.4)	2.6 (0.2)	83 (3.7)	2.2 (0.1)	51 (3.7)	1.6 (0.1)	<0.001
Burgers ^3^	48 (7.7)	1.6 (0.2)	89 (7.4)	2.5 (0.2)	70 (5.0)	2.6 (0.1)	30 (3.0)	1.0 (0.1)	<0.001
Quickbreads	54 (4.7)	1.7 (0.2)	78 (5.9)	2.2 (0.2)	96 (3.9)	2.7 (0.1)	76 (4.4)	2.5 (0.1)	<0.001
Salad dressing	33 (4.5)	1.1 (0.1)	70 (6.2)	2.0 (0.2)	102 (5.8)	2.5 (0.1)	91 (4.5)	2.9 (0.1)	<0.001
Eggs and egg mixed dishes	52 (5.8)	1.7 (0.2)	65 (5.1)	1.6 (0.1)	92 (3.3)	1.9 (0.1)	88 (3.6)	2.8 (0.1)	<0.001
Pork and pork mixed dishes	33 (5.3)	1.1 (0.2)	58 (6.5)	1.6 (0.2)	72 (5.3)	1.5 (0.1)	76 (6.3)	2.4 (0.2)	<0.001
Other white potatoes	38 (4.2)	1.4 (0.1)	43 (4.4)	1.2 (0.1)	56 (3.4)	1.5 (0.1)	72 (6.1)	2.3 (0.2)	<0.001
Other fish and fish mixed dishes	24 (5.4)	0.8 (0.2)	24 (3.6)	0.7 (0.1)	49 (3.2)	1.3 (0.1)	59 (5.4)	1.9 (0.2)	<0.001
**Other ^2^**	568 (12.9)	18.5 (0.4)	617 (19.7)	17.3 (0.4)	807 (19.2)	21.6 (0.4)	765 (13.3)	24.6 (0.3)	-
**Total**	3062 (41.9)	-	3567 (55.6)	-	3742 (37.4)	-	3082 (35.2)	-	-

^1^ Sorted by contribution to 6–11 year-olds; ^2^ Others include all other categories, which contribute <1.3% of total sodium for the total population; ^3^ Burgers, as defined in the database, can only come from quick-service restaurants. All burgers reported from store or full-service restaurants are composed of individual ingredients. Therefore, components of hamburgers/cheeseburgers will be present in the yeast breads, beef and beef mixed dishes, regular cheese and other food groupings.

**Table 2:** Contribution to total sodium intakes from specific food sources by purchase location for children (6–11 years), NHANES 2003–2008.

	Store		Quick-service		Full-service		Other ^1^	
Category ^2^	Average Sodium (SE)	% of total (SE)	Average Sodium (SE)	% of total (SE)	Average Sodium (SE)	% of total (SE)	Average Sodium (SE)	% of total (SE)
Pizza	65 (10.1)	2.1 (0.3)	103 (10.7)	3.4 (0.4)	32 (6.8)	1.0 (0.02)	53 (7.0)	1.7 (0.2)
Yeast breads	175 (8)	5.7 (0.3)	14 (3.1)	0.4 (0.1)	7 (1.8)	0.2 (0.06)	48 (4.8)	1.6 (0.2)
Pasta and pasta dishes	168 (17.7)	5.5 (0.6)	-	-	10 (2.8)	0.3 (0.09)	31 (5.3)	1.0 (0.2)
Chicken and chicken mixed dishes	78 (7.4)	2.5 (0.2)	68 (9.8)	2.2 (0.3)	22 (3.9)	0.7 (0.1)	42 (7.7)	1.4 (0.2)
Sausage, franks, bacon, and ribs	133 (10.4)	4.3 (0.3)	10 (1.8)	0.3 (0.06)	3 (1.0)	0.1 (0.03)	45 (7.1)	1.5 (0.2)
Regular cheese	82 (6.5)	2.7 (0.2)	10 (1.8)	0.3 (0.06)	-	-	23 (3.6)	0.8 (0.1)
Grain-based desserts	82 (5.3)	2.7 (0.2)	5 (1.4)	0.15 (0.04)	2 (0.6)	0.05 (0.02)	28 (3.0)	0.9 (0.1)
Ready-to-eat cereals	93 (5.1)	3.0 (0.2)	-	-	-	-	14 (2.3)	0.5 (0.1)
Beef and beef mixed dishes ^4^	63 (7.3)	2.1 (0.2)	8 (1.6)	0.3 (0.05)	8 (2.1)	0.3 (0.07)	27 (4.2)	0.9 (0.1)
Mexican mixed dishes	41 (10.6)	1.3 (0.3)	32 (11.1)	1.0 (0.04)	9 (3.5)	0.3 (0.01)	22 (6.7)	0.7 (0.2)
Reduced fat milk	57 (4.3)	1.9 (0.1)	-	-	-	-	37 (3.3)	1.2 (0.1)
Soups	80 (9.0)	2.6 (0.3)	-	-	-	-	8 (2.0)	0.3 (0.06)
Potato/corn/other chips	64 (4.6)	2.1 (0.1)	-	-	2 (0.7)^¶^	0.06 (0.02)^¶^	15 (2.1)	0.5 (0.1)
Cold cuts	62 (6)	2.0 (0.2)	-	-	-	-	13 (3.1)	0.4 (0.1)
Pancakes/waffles/French toast	57 (5.8)	1.8 (0.2)	-	-	-	-	8 (2.0)	0.3 (0.06)
Rice and rice mixed dishes	42 (5.6)	1.4 (0.2)	-	-	9 (2.1)	0.3 (0.07)	6 (1.8)	0.2 (0.05)
Condiments	31 (2.6)	1.0 (0.1)	10 (1.2)	0.3 (0.04)	6 (1.3)	0.2 (0.04)	15 (2.3)	0.5 (0.1)
Burgers ^4^	-	-	47 (7.7)	1.5 (0.2)	-	-	-	-
Quickbreads	32 (3.1)	1.1 (0.1)	5 (1.0)	0.2 (0.03)	2 (0.6)	0.06 (0.02)^¶^	14 (2.9)	0.5 (0.1)
Salad dressing	15 (2.3)	0.5 (0.1)	-	-	4 (1.2)	0.14 (0.04)	7 (1.2)	0.2 (0.04)
Eggs and egg mixed dishes	36 (3.8)	1.2 (0.1)	5 (1.5)	0.2 (0.05)	-	-	5 (1.3)	0.2 (0.04)
Pork and pork mixed dishes	20 (2.9)	0.7 (0.1)	-	-	-	-	6 (1.7)	0.2 (0.06)
Other white potatoes	25 (3.7)	0.8 (0.1)	3 (0.7)	0.09 (0.02)	2 (0.7)	0.05 (0.02)	9 (1.5)	0.3 (0.05)
Other fish and fish mixed dishes	16 (3.9)	0.5 (0.1)	-	-	1 (0.2)	0.020 (0.008)	4 (1.6)	0.14 (0.05)
Other ^3^	365 (11.9)	11.9 (0.4)	55 (4.9)	1.8 (0.2)	30 (5.3)	1.0 (0.17)	117 (8.4)	3.8 (0.26)

^1^ Includes school cafeteria, workplace cafeteria, vending machine, gift/from someone else, and other sources; ^2^ Sorted by contribution to 6–11 year-olds; ^3^ Others include all other categories, which contribute <1.3% of total sodium for the total population; ^4^ Burgers, as defined in the database, can only come from quick-service restaurants. All burgers reported from store or full-service restaurants are composed of individual ingredients. Therefore, components of hamburgers/cheeseburgers will be present in the yeast breads, beef and beef mixed dishes, regular cheese and other food groupings. ^¶^ Indicates relative standard error is between 30% and 39.9% of the mean and potentially statistically unreliable.

**Table 3:** Contribution to total sodium intakes from specific food sources by eating location for adolescents (12–19 years), NHANES 2003–2008.

	Store		Quick-service		Full-service		Other ^1^	
Category ^2^	Average Sodium (SE)	% of total (SE)	Average Sodium (SE)	% of total (SE)	Average Sodium (SE)	% of total (SE)	Average Sodium (SE)	% of total (SE)
Pizza	95 (14.2)	2.7 (0.4)	193 (18.4)	5.4 (0.5)	36 (7.1)	1.0 (0.2)	46 (4.9)	1.3 (0.1)
Yeast breads	197 (9.6)	5.5 (0.3)	21 (2.1)	0.6 (0.1)	16 (2.3)	0.4 (0.1)	32 (3.1)	0.9 (0.09)
Pasta and pasta dishes	155 (11.4)	4.3 (0.3)	4 (1.0)	0.12 (0.02)	12 (3.7)	0.32 (0.1)	17 (3.1)	0.5 (0.08)
Chicken and chicken mixed dishes	88 (10.1)	2.5 (0.3)	106 (9.3)	3.0 (0.3)	50 (6.5)	1.4 (0.2)	37 (5.2)	1.0 (0.1)
Sausage, franks, bacon, and ribs	117 (7.8)	3.3 (0.2)	15 (2.1)	0.4 (0.06)	4 (0.7)	0.11 (0.02)	17 (2.7)	0.5 (0.07)
Regular cheese	87 (5.9)	2.4 (0.2)	20 (2.1)	0.6 (0.06)	12 (2.1)	0.32 (0.06)	21 (2.6)	0.6 (0.07)
Grain-based desserts	85 (3.1)	2.4 (0.1)	4 (0.8)	0.1 (0.02)	3 (0.7)	0.07 (0.02)	19 (1.7)	0.5 (0.05)
Ready-to-eat cereals	90 (3.6)	2.5 (0.1)	-	-	-	-	2 (0.4)	0.1 (0.01)
Beef and beef mixed dishes ^4^	85 (6.3)	2.4 (0.2)	25 (3.7)	0.7 (0.1)	20 (4.1)	0.6 (0.1)	19 (2.5)	0.5 (0.07)
Mexican mixed dishes	60 (17.1)	1.7 (0.5)	58 (7.3)	1.6 (0.2)	26 (4.6)	0.7 (0.1)	19 (3.7)	0.5 (0.01)
Reduced fat milk	58 (3.8)	1.6 (0.1)	-	-	-	-	11 (1.7)	0.3 (0.05)
Soups	89 (10.0)	2.5 (0.3)	-	-	-	-	5 (1.8)	0.14 (0.05)
Potato/corn/other chips	82 (4.2)	2.3 (0.1)	2 (0.5)	0.06 (0.01)	2 (0.8)	0.07 (0.02)	11 (1.5)	0.3 (0.04)
Cold cuts	102 (9.4)	2.9 (0.3)	8 (2.3)	0.2 (0.06)	2 (0.7)	0.05 (0.02)	13 (2.7)	0.4 (0.07)
Pancakes/waffles/French toast	34 (4.3)	0.9 (0.1)	2 (0.5)	0.04 (0.01)	4 (1.6)	0.11 (0.04)	4 (1.0)	0.1 (0.03)
Rice and rice mixed dishes	48 (5.9)	1.4 (0.1)	8 (1.2)	0.2 (0.03)	16 (2.8)	0.4 (0.07)	7 (1.6)	0.2 (0.04)
Condiments	35 (2.9)	1.0 (0.1)	20 (2.3)	0.6 (0.06)	19 (6.0)	0.5 (0.16)	18 (2.4)	0.5 (0.07)
Burgers ^4^	-	-	87 (7.4)	2.4 (0.2)	-	-	-	-
Quickbreads	42 (3.6)	1.2 (0.1)	17 (4.2)	0.5 (0.01)	8 (2.3)	0.2 (0.06)	11 (1.4)	0.3 (0.04)
Salad dressing	38 (4.6)	1.1 (0.1)	8 (1.4)	0.2 (0.04)	14 (3.0)	0.4 (0.08)	10 (1.8)	0.3 (0.05)
Eggs and egg mixed dishes	40 (3.4)	1.1 (0.1)	14 (2.9)	0.4 (0.08)	8 (2.7)	0.23 (0.07)	3 (0.9)	0.08 (0.26)
Pork and pork mixed dishes	33 (4.2)	0.9 (0.1)	7 (2.1)	0.2 (0.06)	-	-	6 (1.3)	0.2 (0.04)
Other white potatoes	23 (3.3)	0.6 (0.1)	5 (1.3)	0.15 (0.04)	7 (2.2)	0.2 (0.06)	8 (2.5)	0.2 (0.07)
Other fish and fish mixed dishes	10 (2.4)	0.3 (0.07)	8 (2.7)	0.23 (0.08)	2 (0.5)	0.1 (0.01)	4 (1.2)	0.12 (0.03)
Other ^3^	405 (16.0)	11.4 (0.4)	74 (7.6)	2.1 (0.2)	42 (4.7)	1.2 (0.1)	96 (7.5)	2.7 (0.21)

^1^ Includes school cafeteria, workplace cafeteria, vending machine, gift/from someone else, and other sources; ^2^ Sorted by contribution to 6–11 year-olds; ^3^ Others include all other categories, which contribute <1.3% of total sodium for the total population; ^4^ Burgers, as defined in the database, can only come from quick-service restaurants. All burgers reported from store or full-service restaurants are composed of individual ingredients. Therefore, components of hamburgers/cheeseburgers will be present in the yeast breads, beef and beef mixed dishes, regular cheese and other food groupings. ^¶^ Indicates relative standard error is between 30% and 39.9% of the mean and potentially statistically unreliable.

**Table 4:** Contribution to total sodium intakes from specific food sources by eating location for adults (20–50 years), NHANES 2003–2008.

	Store		Quick-service		Full-service		Other^ 1^	
Category^ 2^	Average Sodium (SE)	% of total (SE)	Average Sodium (SE)	% of total (SE)	Average Sodium (SE)	% of total (SE)	Average Sodium (SE)	% of total (SE)
Pizza	56 (6.2)	1.5 (0.2)	138 (12)	3.7 (0.3)	35 (4.3)	0.9 (0.1)	10 (2.5)	0.3 (0.07)
Yeast breads	189 (5.5)	5.1 (0.2)	25 (1.8)	0.7 (0.05)	29 (2.1)	0.8 (0.05)	27 (2.3)	0.7 (0.06)
Pasta and pasta dishes	140 (9.3)	3.7 (0.2)	6 (1.0)	0.2 (0.03)	17 (2.2)	0.5 (0.06)	18 (3.3)	0.5 (0.09)
Chicken and chicken mixed dishes	102 (5.5)	2.7 (0.1)	99 (6.0)	2.6 (0.2)	56 (4.1)	1.5 (0.1)	17 (2.1)	0.5 (0.06)
Sausage, franks, bacon, and ribs	127 (7.5)	3.4 (0.2)	16 (1.6)	0.4 (0.1)	14 (1.6)	0.4 (0.04)	21 (2.3)	0.6 (0.1)
Regular cheese	92 (4)	2.5 (0.1)	24 (2.3)	0.7 (0.1)	14 (1.4)	0.4 (0.04)	16 (1.2)	0.4 (0.03)
Grain-based desserts	78 (3.6)	2.1 (0.1)	3 (0.6)	0.1 (0.02)	5 (0.7)	0.1 (0.02)	23 (1.6)	0.6 (0.04)
Ready-to-eat cereals	55 (2.3)	1.5 (0.1)	-	-	-	-	1 (0.2)	0.015 (0.04)
Beef and beef mixed dishes ^4^	100 (5)	2.7 (0.1)	28 (2.8)	0.8 (0.1)	29 (2.3)	0.8 (0.06)	17 (1.6)	0.5 (0.04)
Mexican mixed dishes	56 (5.7)	1.5 (0.1)	70 (8.2)	1.9 (0.2)	28 (3.8)	0.7 (0.1)	18 (2.2)	0.5 (0.06)
Reduced fat milk	39 (2.3)	1.0 (0.1)	-	-	1 (0.2)	0.02 (0.001)	2 (0.4)	0.1 (0.01)
Soups	91 (6.7)	2.4 (0.2)	6 (1.3)	0.2 (0.03)	19 (2.7)	0.5 (0.07)	11 (2.5)	0.3 (0.07)
Potato/corn/other chips	52 (2.4)	1.4 (0.1)	3 (0.5)	0.1 (0.01)	3 (0.3)	0.1 (0.009)	7 (0.6)	0.2 (0.02)
Cold cuts	97 (6)	2.6 (0.02)	14 (2.1)	0.4 (0.06)	8 (1.9)	0.2 (0.05)	11 (1.7)	0.3 (0.05)
Pancakes/waffles/French toast	21 (2.1)	0.6 (0.06)	2 (0.4)	0.04 (0.01)	6 (1.1)	0.15 (0.03)	2 (0.4)	0.04 (0.01)
Rice and rice mixed dishes	70 (5.8)	1.9 (0.2)	16 (2.8)	0.4 (0.1)	33 (2.7)	0.9 (0.07)	9 (1.6)	0.2 (0.04)
Condiments	37 (2.5)	1.0 (0.1)	19 (1.7)	0.5 (0.1)	18 (1.8)	0.5 (0.05)	9 (1.2)	0.2 (0.03)
Burgers^ 4^	-	-	68 (5.0)	1.8 (0.1)	-	-	-	-
Quickbreads	60 (3.1)	1.6 (0.1)	15 (1.4)	0.4 (0.04)	12 (1.5)	0.3 (0.04)	10 (1.1)	0.3 (0.3)
Salad dressing	50 (4.2)	1.3 (0.1)	13 (1.5)	0.3 (0.04)	29 (2.8)	0.8 (0.07)	10 (1.2)	0.3 (0.3)
Eggs and egg mixed dishes	49 (2)	1.3 (0.1)	19 (2.3)	0.5 (0.1)	15 (2)	0.4 (0.05)	10 (1.4)	0.3 (0.04)
Pork and pork mixed dishes	43 (4.1)	1.2 (0.1)	7 (1.4)	0.2 (0.04)	10 (1.7)	0.3 (0.04)	11 (2.1)	0.3 (0.05)
Other white potatoes	29 (1.7)	0.8 (0.05)	6 (0.7)	0.2 (0.02)	10 (1.2)	0.3 (0.03)	11 (1.8)	0.3 (0.05)
Other fish and fish mixed dishes	24 (2.6)	0.6 (0.07)	9 (1.8)	0.2 (0.05)	10 (1.6)	0.3 (0.04)	6 (1.0)	0.2 (0.03)
Other ^3^	518 (12.8)	13.9 (0.3)	85 (5.8)	2.3 (0.14)	100 (7.5)	2.7 (0.2)	103 (4.9)	2.8 (0.13)

^1^ Includes school cafeteria, workplace cafeteria, vending machine, gift/from someone else, and other sources; ^2^ Sorted by contribution to 6–11 year-olds; ^3^ Others include all other categories, which contribute <1.3% of total sodium for the total population; ^4^ Burgers, as defined in the database, can only come from quick-service restaurants. All burgers reported from store or full-service restaurants are composed of individual ingredients. Therefore, components of hamburgers/cheeseburgers will be present in the yeast breads, beef and beef mixed dishes, regular cheese and other food groupings.

**Table 5:** Contribution to total sodium intakes from specific food sources by eating location for adults (≥51 years), NHANES 2003–2008.

	Store		Quick-Service		Full-service		Other ^1^	
Category ^2^	Average Sodium (SE)	% of total (SE)	Average Sodium (SE)	% of total (SE)	Average Sodium (SE)	% of total (SE)	Average Sodium (SE)	% of total (SE)
Pizza	28 (4.8)	0.9 (0.2)	44 (8.0)	1.4 (0.26)	16 (3.2)	0.5 (0.10)	4 (1.3)^¶^	0.1 (0.04)
Yeast breads	221 (5.4)	7.2 (0.2)	14 (1.4)	0.4 (0.05)	27 (2.4)	0.9 (0.08)	26 (2.1)	0.8 (0.07)
Pasta and pasta dishes	89 (7.9)	2.9 (0.3)	2 (1.1)	-	-	0.4 (0.07)	16 (2.3)	0.5 (0.08)
Chicken and chicken mixed dishes	74 (5)	2.4 (0.2)	42 (5.1)	1.4 (0.17)	38 (3.8)	1.2 (0.13)	16 (2.2)	0.5 (0.07)
Sausage, franks, bacon, and ribs	112 (7)	3.6 (0.2)	14 (2.4)	0.5 (0.08)	13 (1.8)	0.4 (0.06)	19 (2.5)	0.6 (0.08)
Regular cheese	80 (4.9)	2.6 (0.1)	9 (1.2)	0.3 (0.04)	8 (1.1)	0.3 (0.04)	11 (1.5)	0.4 (0.05)
Grain-based desserts	81 (3.9)	2.6 (0.1)	2 (0.5)	0.1 (0.01)	5 (0.7)	0.2 (0.02)	26 (2.2)	0.8 (0.07)
Ready-to-eat cereals	61 (2.4)	2.0 (0.1)	-	-	-	-	2 (0.4)	0.1 (0.01)
Beef and beef mixed dishes ^4^	86 (6.4)	2.8 (0.2)	16 (2.7)	0.5 (0.09)	27 (2.7)	0.9 (0.09)	19 (2.5)	0.6 (0.08)
Mexican mixed dishes	33 (4.3)	1.1 (0.1)	17 (2.9)	0.6 (0.10)	20 (4.3)	0.6 (0.14)	7 (1.4)	0.2 (0.05)
Reduced fat milk	36 (1.7)	1.2 (0.1)	-	-	-	-	2 (0.4)	0.1 (0.02)
Soups	102 (7.6)	3.3 (0.2)	3 (0.8)	0.1 (0.02)	24 (3.4)	0.8 (0.11)	14 (2.5)	0.4 (0.08)
Potato/corn/other chips	32 (1.6)	1.0 (0.1)	0 (0.1)	0.01 (0.002)	4 (0.6)	0.1 (0.02)	3 (0.4)	0.1 (0.01)
Cold cuts	87 (4.2)	2.8 (0.1)	7 (1.5)	0.2 (0.05)	5 (1.4)	0.1 (0.04)	9 (2.1)	0.3 (0.07)
Pancakes/waffles/French toast	21 (3.7)	0.7 (0.1)	1 (0.3)	0.02 (0.001)	7 (1.6)	0.2 (0.05)	-	-
Rice and rice mixed dishes	48 (4.8)	1.6 (0.2)	6 (1.5)	0.2 (0.05)	18 (2.1)	0.6 (0.07)	5 (1.2)	0.2 (0.04)
Condiments	24 (2.1)	0.8 (0.07)	8 (0.9)	0.3 (0.03)	15 (2.3)	0.5 (0.07)	4 (0.5)	0.1 (0.01)
Burgers ^4^	-	-	30 (3.0)	1.0 (0.10)	-	-	-	-
Quickbreads	49 (3.7)	1.6 (0.1)	11 (1.7)	0.4 (0.06)	9 (1.2)	0.3 (0.04)	7 (1)	0.2 (0.03)
Salad dressing	49 (3.2)	1.6 (0.1)	7 (1.4)	0.2 (0.04)	26 (2.8)	0.9 (0.09)	9 (1.3)	0.3 (0.04)
Eggs and egg mixed dishes	51 (2.8)	1.7 (0.1)	12 (2.1)	0.4 (0.07)	16 (2.1)	0.5 (0.07)	8 (1.5)	0.3 (0.05)
Pork and pork mixed dishes	48 (4.8)	1.6 (0.2)	5 (1.5)^¶^	0.15 (0.05)^¶^	11 (2.7)	0.4 (0.09)	12 (2.2)	0.4 (0.07)
Other white potatoes	43 (6.2)	1.4 (0.2)	5 (0.9)	0.2 (0.03)	13 (1.5)	0.4 (0.05)	12 (1.4)	0.4 (0.05)
Other fish and fish mixed dishes	23 (2.2)	0.7 (0.07)	10 (2.9)	0.3 (0.10)	11 (2.1)	0.4 (0.07)	15 (3.7)	0.5 (0.12)
Other ^3^	547 (13.3)	17.7 (0.4)	40 (4.0)	1.3 (0.1)	82 (5.4)	2.7 (0.2)	96 (5.5)	3.1 (0.17)

^1^ Includes school cafeteria, workplace cafeteria, vending machine, gift/from someone else, and other sources; ^2^ Sorted by contribution to 6–11 year-olds; ^3^ Others include all other categories, which contribute <1.3% of total sodium for the total population; ^4^ Burgers, as defined in the database, can only come from quick-service restaurants. All burgers reported from store or full-service restaurants are composed of individual ingredients. Therefore, components of hamburgers/cheeseburgers will be present in the yeast breads, beef and beef mixed dishes, regular cheese and other food groupings. ^¶^ Indicates relative standard error is between 30% and 39.9% of the mean and potentially statistically unreliable.

## 4. Discussion

The present analyses of nationally representative NHANES 2003–2008 data provide important insight into sodium intakes by age group, race/ethnicity, food location of origin and specific food source. The parallel evaluation of food location of origin (e.g., store and QSR) and food sources (e.g., pizza and yeast breads) by age group is novel and enhances approaches of dietary surveillance, while also providing context to potential population-wide interventions. 

Food locations of origin were derived from the current classification of NHANES data, whereas specific food sources were based on the National Cancer Institute food classification scheme. The NCI scheme was featured prominently in the 2010 Dietary Guidelines for Americans (DGA) [13] to identify the major sources of sodium and other nutrients in the American diet. This facilitates comparison of the results presented here, with those in the 2010 DGAs.

Dietary Guidelines 2010 have noted that substantial amounts of dietary sodium come from restaurant and processed foods [20]. In other studies, FAFH meals were further associated with high sodium content and lower nutrient quality [6,7], particularly on a per-calorie basis. For those reasons, many efforts to reduce the amount of sodium in the American diet have primarily focused on foods purchased and consumed away from home, particularly in restaurants [11,21].

The present analyses, based on nationally representative data from 2003 to 2008, provide a clear indication of the major sources of sodium in the American diet, together with their purchase locations. These data can shape and inform public health policies aimed at reducing the sodium content of the diet by age group.

First, foods purchased in stores contributed the bulk of dietary sodium for all age groups (58%–65%), consistent with the observations of the 2010 Dietary Guidelines [13], Centers for Disease Control and Prevention [22] and the Institute of Medicine [23]. Restaurants, including QSR and FSR, contributed a maximum of about 30% of dietary sodium. Of these, QSR foods accounted for up to 19.9% of sodium in the diets of adolescents (12–19) and 18.5% of sodium in the diets of adults (20–50). The contribution of restaurant foods to sodium intakes declined sharply with age, as the contribution of store-bought foods increased. School meals accounted for up to 10.4% of sodium in the diets of children and 6% in the diets of adolescents. 

The present analyses of purchase location by specific food source, conducted for each age group, are unique. They show the purchase source and the sodium contribution of foods that are often viewed as problematic. Once the frequency of consumption was taken into account, it became clear that the top sources of sodium in the diet were not necessarily the most sodium-rich foods. Rather, more sodium came from frequently eaten items such as bread, pizza and pasta, chicken dishes, processed meats, beef, cheese, cold cuts and even desserts. QSR-derived pizza, burgers, chicken dishes, and Mexican foods individually contributed smaller amounts that added up to a maximum ranging from about 4% to 12%, depending on age. The present results are consistent with past analyses of the major sodium food sources in Australia [16], Canada [24], France [25] and the US [22].

In particular, the present results echo the prior findings of the CDC [22], based on 7,227 participants aged ≥2 years in the 2007–2008 NHANES. That study also analyzed population proportions of sodium consumption by location of origin and food source but did not provide a breakdown by age. In the previous CDC study, approximately 44% of dietary sodium came from 10 food categories: bread and rolls, cold cuts/cured meats, pizza, poultry, soups, sandwiches, cheese, pasta mixed dishes, meat mixed dishes, and savory snacks. For most of these categories, >70% of the sodium consumed came from foods obtained at a store, consistent with the present results. For pizza and chicken dishes, 51% and 27% of sodium respectively came from foods obtained at QSR as opposed to stores. Importantly, mean sodium consumption per calorie consumed was significantly greater for foods and beverages obtained from restaurants *vs.* stores, again consistent with the present results [22]. 

The key contribution of this study is the specific quantification of the location of origin and food source for sodium purchased among four distinct age groups. This research builds upon prior CDC analysis by evaluating sources and location of origin within specific age groups, an important step in better understanding the potential for interventions [22]. For example, while the CDC report notes that 1.7%–8.2% of sodium comes from pizza overall depending on age [22], our analysis shows that 5.4% of sodium among adolescents comes from QSR, compared to only 1.4% among older adults. This adds important information that may be useful in terms of understanding the potential impact of interventions that target specific sub-populations or food purchasing locations. For example, an effort to reduce sodium intake among older adults would necessitate a very different strategy in terms of both food sources and locations targeted than a strategy tailored for the adolescent population. 

Previous analyses, including the CDC report [22], have evaluated food sources of sodium by race/ethnicity, but to our knowledge this is the first analysis of food location of origin by race/ethnicity. First, it is notable that for all race/ethnicity groups, sodium obtained from stores alone exceeded the recommended intake of 1500 mg/day for select populations, and came close to approaching the recommended population intake amount of 2300 mg/day. However, non-Hispanic black and Mexican-American/other Hispanic adults obtained significantly less sodium from stores than non-Hispanic whites. Non-Hispanic black adults obtained significantly more sodium from QSR than non-Hispanic whites, though they obtained less from FSR. Mexican-American/other Hispanic adults also obtained significantly less sodium from FSR than non-Hispanic Whites. While on a per-calorie basis, more sodium is obtained from QSR/FSR than from the store [22], these data suggest that interventions to reduce sodium intakes among adults for all race/ethnicity groups must engage intakes from all sources, including stores. 

Studies based on analyses of NHANES data share many of the same limitations. First, the increasingly important category of store prepared foods could not be taken into account and would be misclassified here. Second, a single 24-h recall is not necessarily representative of a habitual diet of individuals, though it is an adequate tool for identifying the habits of large populations. Third, there are many ways of aggregating individual foods into food groups and food categories. The present option was chosen since it was developed by a federal agency and was presented as part of the 2010 Dietary Guidelines. In addition, there may be error present in the coding/grouping of foods. However, such error is likely to be minimal, as misclassification of frequently consumed foods is less likely than for less frequently consumed foods. Fourth, the present categorization did not distinguish between purchase location and actual eating location: home, work, car, public transport, or other. More work is needed on how individuals interact with their food environment in both space and time. Lastly, the lack of data on salt added at the table, which is not captured by the 24-h recall, may introduce additional bias and could potentially lead to an under-estimate of salt added at the table for both store-bought and restaurant foods. However, the NHANES 24-h recall data does account for salt added during cooking. 

## 5. Conclusions

The present analyses represent the first demonstration of how sodium intakes are partitioned by location and source and provide important information about dietary habits by age group and race/ethnicity. Future analyses, utilizing the same data and approach, could provide data by family income, education, place of birth or any other variable measured in the NHANES data. Although the majority of sodium consumption was derived from foods purchased from stores and prepared at home, almost one third of total sodium came from restaurant foods. One frequently expressed concern has been that Americans do not compensate for away from home foods by making healthier food choices at home [7]. Reducing sodium intake at the population-level will require modifying food purchase behaviors at both stores and restaurants. 

## Implications

Food manufacturers, supermarkets and grocery stores, restaurants and schools can all implement changes that would make a meaningful contribution to reducing the amount of sodium in the American diet.

## Supplementary Files

Supplementary File 1 Supplementary Information (PDF, 67 KB)
