# Supplementary material for: Sodium Intakes of US Children and Adults from Foods and Beverages by Location of Origin and by Specific Food Source
Source: Nutrients. 2013 May 28;5(6):1840–55. doi: 10.3390/nu5061840 (PMC3725480; doi:10.3390/nu5061840)
Supplement: Supplementary File 1 — Supplementary Information (PDF, 67 KB) [file nutrients-05-01840-s001.pdf]

Supplementary Information

Table S1. Proportion of total sodium by category and source for children (age 6–11 years).

Category and Source of Sodium	% of Total [1]
Yeast breads-Store	5.7
Pasta and pasta dishes-Store	5.5
Sausage, franks, bacon, and ribs-Store	4.3
Pizza-QSR	3.4
Ready-to-eat cereals-Store	3.0
Grain-based desserts-Store	2.7
Regular cheese-Store	2.7
Soups-Store	2.6
Chicken and chicken mixed dishes-Store	2.5
Chicken and chicken mixed dishes-QSR	2.2
Pizza-Store	2.1
Potato/corn/other chips-Store	2.1
Beef and beef mixed dishes-Store	2.1
Cold cuts-Store	2.0
Reduced fat milk-Store	1.9
Pancakes/waffles/French toast-Store	1.8
Burgers-QSR	1.5
Rice and rice mixed dishes-Store	1.4
Mexican mixed dishes-Store	1.3
Pizza-School	1.3
Crackers-Store	1.2
Eggs and egg mixed dishes-Store	1.2
Reduced fat milk-School	1.1
Quickbreads-Store	1.1
Chicken and chicken mixed dishes-School	1.0
Mexican mixed dishes-QSR	1.0
Pizza-FSR	1.0
Condiments-Store	1.0
Whole milk-Store	1.0
Others [2]	38.1
Others-not store, QSR, FSR or school	9.1

[1] May not sum to 100 due to rounding; [2] Less than 1% of total sodium.

Table S2. Proportion of total sodium by category and source for adolescents (age 12–19 years).

**Category and Source of Sodium**	**% of Total** [1]
Yeast breads-Store	5.5
Pizza-QSR	5.4
Pasta and pasta dishes-Store	4.3
Sausage, franks, bacon, and ribs-Store	3.3
Chicken and chicken mixed dishes-QSR	3.0
Cold cuts-Store	2.9
Pizza-Store	2.7
Ready-to-eat cereals-Store	2.5
Soups-Store	2.5
Chicken and chicken mixed dishes-Store	2.5
Regular cheese-Store	2.4
Burgers-QSR	2.4
Beef and beef mixed dishes-Store	2.4
Grain-based desserts-Store	2.4
Potato/corn/other chips-Store	2.3
Mexican mixed dishes-Store	1.7
Mexican mixed dishes-QSR	1.6
Reduced fat milk-Store	1.6
Chicken and chicken mixed dishes-FSR	1.4
Rice and rice mixed dishes-Store	1.4
Soda, energy and sports drinks-Store	1.3
Quickbreads-Store	1.2
Eggs and egg mixed dishes-Store	1.1
Salad dressing-Store	1.1
Pizza-FSR	1.0
Pizza-School	1.0
Condiments-Store	1.0
Crackers-Store	1.0
Others [2]	31.0
Others-not store, QSR or FSR	6.3

[1] May not sum to 100 due to rounding; [2] Less than 1% of total sodium.

Table S3. Proportion of total sodium by category and source for adults (age 20–50 years).

**Category and Source of Sodium**	**% of Total** [1]
Yeast breads-Store	5.1
Pasta and pasta dishes-Store	3.7
Pizza-QSR	3.7
Sausage, franks, bacon, and ribs-Store	3.4
Chicken and chicken mixed dishes-Store	2.7
Beef and beef mixed dishes-Store	2.7
Chicken and chicken mixed dishes-QSR	2.6
Cold cuts-Store	2.6
Regular cheese-Store	2.5
Soups-Store	2.4
Grain-based desserts-Store	2.1
Mexican mixed dishes-QSR	1.9
Rice and rice mixed dishes-Store	1.9
Burgers-QSR	1.8
Quickbreads-Store	1.6
Mexican mixed dishes-Store	1.5
Chicken and chicken mixed dishes-FSR	1.5
Pizza-Store	1.5
Ready-to-eat cereals-Store	1.5
Potato/corn/other chips-Store	1.4
Salad dressing-Store	1.3
Eggs and egg mixed dishes-Store	1.3
Pork and pork mixed dishes-Store	1.2
Soda, energy and sports drinks-Store	1.1
Reduced fat milk-Store	1.0
Condiments-Store	1.0
Others [2]	45.1

[1] May not sum to 100 due to rounding; [2] Less than 1% of total sodium.

Table S4. Proportion of total sodium by category and source for adults (age ≥51 years).

**Category and Source of Energy**	**% of Total** [1]
Yeast breads-Store	7.1
Sausage, franks, bacon, and ribs-Store	3.7
Soups-Store	3.3
Pasta and pasta dishes-Store	2.8
Beef and beef mixed dishes-Store	2.8
Cold cuts-Store	2.7
Grain-based desserts-Store	2.6
Regular cheese-Store	2.5
Chicken and chicken mixed dishes-Store	2.4
Ready-to-eat cereals-Store	1.9
Quickbreads-Store	1.6
Eggs and egg mixed dishes-Store	1.6
Rice and rice mixed dishes-Store	1.6
Salad dressing-Store	1.6
Pork and pork mixed dishes-Store	1.5
Pizza-QSR	1.4
Chicken and chicken mixed dishes-QSR	1.4
Other white potatoes-Store	1.3
Chicken and chicken mixed dishes-FSR	1.3
Crackers-Store	1.2
Mexican mixed dishes-Store	1.2
Reduced fat milk-Store	1.2
Potato/corn/other chips-Store	1.0
Burgers-QSR	1.0
Nuts/seeds and nut/seed mixed dishes-Store	1.0
Others [2]	48.1

[1] May not sum to 100 due to rounding; [2] Less than 1% of total energy.

© 2013 by the authors; licensee MDPI, Basel, Switzerland. This article is an open access article distributed under the terms and conditions of the Creative Commons Attribution license (http://creativecommons.org/licenses/by/3.0/).
